# Possible step-up in prevalence for *Escherichia coli* ST131 from fecal to clinical isolates: inferred virulence potential comparative studies within phylogenetic group B2

**DOI:** 10.1186/s12929-022-00862-7

**Published:** 2022-10-07

**Authors:** Timothy Kudinha, Fanrong Kong

**Affiliations:** 1 NSW Health Pathology, Regional and Rural, Orange Hospital, Orange, NSW Australia; 2 School of Biomedical Sciences, Charles Sturt University, Leeds Parade, Orange, NSW 2800 Australia; 3 NSW Health Pathology, CIDMLS, Westmead Hospital, Westmead, NSW 2145 Australia

**Keywords:** *Escherichia coli*, Virulence genes, Antibiotic resistance

## Abstract

**Background:**

*Escherichia coli* sequence type (ST)131 is an important urinary tract pathogen, and is responsible for considerable healthcare-associated problems and costs worldwide. A better understanding of the factors that contribute to its rapid worldwide spread may help in arresting its continual spread. We studied a large collection of fecal and urinary *E. coli* ST131 and *E. coli* non-ST131 phylogenetic group B2 isolates, from women, men and children, in regional NSW, Australia.

**Results:**

We found out that there was a step up in ST131 prevalence (and possibly in virulence) from fecal to clinical (urinary) isolates in general, and specifically among ciprofloxacin resistant isolates, in the 3 host groups. Furthermore, our results revealed that the inferred virulence potential of the ST131 isolates (as measured by VF gene scores) was much higher than that of non-ST131 phylogenetic group B2 isolates, and this was much more pronounced amongst the urinary isolates. This finding suggests presence of possible *E. coli* phylogenetic B2 subgroups with varying levels of virulence, with ST131 being much more virulent compared to others. A strong association between ST131 and fluoroquinolone (FQ) resistance was also demonstrated, suggesting that FQ use is related to ST131 emergence and spread. Specifically, about 77% of ST131 isolates from women and men, and 47% from children, were extended spectrum β- lactamase (ESBL) producers. Moreover, FQ resistant ST131 ESBL isolates on average harbored more VF genes than all other isolates.

**Conclusions:**

The strong association between ST131 prevalence and FQ resistance amongst the studied isolates suggests that FQ use is related to ST131 emergence and spread. Furthermore, our results demonstrate that FQ resistance and a plurality of VF genes can exist together in ST131, something that has traditionally been regarded as being inversely related. This may partly contribute to the emergence and worldwide spread of ST131.

**Supplementary Information:**

The online version contains supplementary material available at 10.1186/s12929-022-00862-7.

## Introduction

*Escherichia coli* causes several extra-intestinal infections, including urinary tract infection (UTI), bacteremia, and meningitis, among several others. This organism is responsible for considerable UTI morbidity, mortality and healthcare associated costs, worldwide [1]. *E. coli* possess a wide variety of virulence factors (VFs) that help the organism to infect and damage the host. Hence most studies on UTI pathogenesis have targeted this organism and associated VFs in an effort to gain further insights that could contribute to better management of UTIs.

*Escherichia coli* sequence type 131 (ST131) has recently emerged as an important UTI public health pathogen due to its rapid spread worldwide and multi-drug resistance, presenting challenges for management of infections caused by this organism. Several studies have been carried out to unravel bacterial characteristics of the organism responsible for its rapid spread worldwide, with most studies focusing on the role played by VFs in UTI pathogenesis. However, studies on ST131 directed at elucidating pathogenesis based on VF genes have yielded conflicting results, with some studies suggesting that ST131 is as, or less virulent than other uropathogenic *E. coli* (UPEC) strains, whilst other studies yielded contrary findings [2].

Most VF gene comparative studies on ST131 vs. non-ST131 strains did not take into consideration the possible confounding effect of phylogenetic groups on VF gene distribution. Since ST131 is from phylogenetic group B2, it is important that in trying to understand the virulence potential of ST131, comparisons must be made in relation to non-ST131 B2 isolates so as to eliminate the possible phylogenetic group B2 confounding effect. This may help identify specific factors that are unique to ST131, which may give some insight into its rapid worldwide spread. Furthermore, some studies suggest that the unique combination of antibiotic resistance and specific VFs promote the rapid spread of ST131 [3, 4]. However, few studies have adequately assessed this finding against antimicrobial resistant isolates specifically within phylogenetic group B2. Furthermore, there is a scarcity of data on whether ST131 has a bigger step-up in prevalence from fecal to UTI isolates than do other fluoroquinolone (FQ) resistant isolates, specifically within group B2. Accordingly, we studied the VF gene distribution of ST131 vs. non-ST131 B2 isolates, as well as FQ susceptibility of ST131 fecal and UTI isolates from women, men and children in the Central West region of NSW, Australia.

## Materials and methods

### Bacterial isolates

We have previously carried out extensive work on *E. coli* UTI and fecal isolates from women, men and children, in the Central West region of New South Wales (NSW) in Australia, creating a large pool of well characterized isolates, collected over a 3-year period (June 2009-July 2012). The isolates for this study were obtained from this pool. The *E. coli* isolates were identified by both conventional biochemical and molecular tests, and preserved at − 80 °C in glycerol broth until further use, and only one isolate per study subject was included.

Urine specimens (midstream and clean catch) were collected according to a guiding protocol, from women, men and children, presenting to randomly selected participating centers. To allow a focus on bacterial characteristics, with minimal confounding by other host characteristics, patients with known diabetes mellitus, diarrhoea, antibiotic therapy in the last month, menstruation, or urinary tract abnormalities, were excluded. A diagnosis of cystitis or pyelonephritis was based on specific clinical manifestations, plus a urine culture yielding ≥ 10^8^ cfu/L of *E. coli*. Cystitis was defined by presence of urinary frequency, dysuria, and/or suprapubic tenderness, without fever or loin pain. Pyelonephritis was defined by presence of urinary symptoms, fever of ≥ 38 °C, and flank pain, with or without nausea and/or vomiting. During the same time period, rectal swabs were collected from consenting women, men, and children of consenting parents/guardians, who, according to self-report, lacked UTI-associated manifestations. Ten arbitrarily chosen *E. coli* isolates were picked from each rectal swab culture and tested for ST131 status. A previous study indicated that the chance of an arbitrarily chosen isolate representing the quantitative dominant clone in a culture is 86% [5].

From the above collections, all the ST131 and non-ST131 phylogenetic group B2 isolates from feces and UTI cases, from children, women, men and children, collected in the same geographical location and time period, were studied. As much as possible, the fecal isolates were matched to the UTI isolates for age, sex and geographical location.

### Antimicrobial susceptibility testing.

The studied *E. coli* isolates were tested for susceptibilities to the following antibiotics according to the disc diffusion method as specified by the Clinical and Laboratory Standards Institute (CLSI, M02-A12) [6]; amikacin (30 µg), amoxicillin-clavulanate (60 µg), ampicillin (25 µg), ceftazidime (30 µg), ceftriaxone (30 µg), cephalothin (30 µg), ciprofloxacin (10 µg), imipenem (10 µg), nitrofurantoin (300 µg), and trimethoprim-sulfamethoxazole (5 µg). ESBL detection was performed by using the double disk-synergy test between clavulanic acid and extended-spectrum cephalosporins (ceftazidime and cefotaxime) on Mueller–Hinton agar, as previously described [7].

### Molecular methods

*Escherichia coli* phylogenetic grouping was determined as previously described [8], with group B2 isolates further typed for ST131 status by PCR-based detection of ST131-specific single nucleotide polymorphisms (SNPs) in the *mdh* and *gyrB* genes [9, 10]. PCR-based O (somatic antigen) typing was used to detect the 2 ST131-associated O types, O16 and O25 [9]. The O25 isolates were further characterized as O25a versus O25b by variant-specific PCR [11, 12]. Typing for 22 VF genes (see Table 2 for full list), was done by multiplex PCR reverse line blot (mPCR-RLB) assay as described before [13]. VF gene score for an isolate was the sum of VF genes, with multiple *pap* operons counted as one factor. Such molecular characteristics have been shown to predict in vivo virulence [14].

### Statistical analysis

Proportions were compared using Fishers exact test, whilst VF scores were tested by the Mann–Whitney U test. P values of < 0.05 were considered statistically significant.

## Results

### Distribution of ST131 among UTI and fecal isolates by source, ciprofloxacin susceptibility phenotype and B2 status

A total of 1367 *E. coli* fecal isolates from women (n = 458), children (n = 508), and men (n = 401) were studied alongside 1416 UTI (cystitis and pyelonephritis) isolates (women = 623; children = 395; men = 389) (Table 1). Overall, for each host group, ST131 prevalence was significantly higher in UTI isolates (at least double) than in fecal isolates (P < 0.001). For fecal isolates, ST131 prevalence ranged from 2% in men (8 of 401) to 3% (14 of 508) in children, and finally 5% (23 of 458) in women. In contrast to fecal isolates, ST131 prevalence in UTI isolates followed a significant gradient, being least in children (8%), rising to 13% in men, and finally highest in women (20%) (P < 0.001).

**Table 1:** Prevalence of ST131 among *Escherichia coli* isolates in relation to source, ciprofloxacin phenotype, and phylogenetic group

Host group	Isolate subgroup	Prevalence of ST131, proportion (%)		P value^c^
		Fecal^a^	Clinical^b^	
Children	Total population	14/508 (3)	32/395 (8)	0.02
	Ciprofloxacin-resistant	4/17 (24)	15/32 (47)	< 0.001
	Group B2	14/106 (13)	32/237 (14)	NS^d^
Women	Total population	23/458 (5)	124/623 (20)	< 0.001
	Ciprofloxacin-resistant	7/30 (23)	65/83 (78)	< 0.001
	Group B2	23/78 (29)	124/398 (31)	NS
Men	Total population	8/401 (2)	49/389 (13)	< 0.001
	Ciprofloxacin-resistant	5/14 (35)	27/35 (77)	< 0.001
	Group B2	8/64 (13)	49/241 (20)	0.021

^a^Fecal isolates from healthy subjects, matched by age, time, and location to the clinical isolates

^b^Urine isolates from patients with cystitis

^c^P values (by Fisher’s exact test) are show where P < 0.05

NS^d^: Not significant; P values > 0.05

In each host group, the proportion of ST131 isolates resistant to ciprofloxacin was significantly higher in UTI (almost double) than fecal isolates, with the difference much more pronounced in isolates from women, where the prevalence of ciprofloxacin resistance in UTI isolates was more than treble that of fecal isolates (P < 0.001) (Table 1). Furthermore, among UTI isolates, a lower proportion (47%) of ST131 isolates from children were ciprofloxacin resistant compared to at least 77% in adults (P < 0.001).

For fecal isolates, the prevalence of ciprofloxacin resistance was similar in children and women (about 23%), which was significantly lower than that in men (35%) (P = 0.01). In children and women, ST131 isolates comprised about 14% and 30% of phylogenetic group B2 isolates, respectively, with no significant difference between the proportions in both fecal and UTI isolates. However, in men, ST131 isolates comprised 20% of B2 UTI isolates, which was significantly different to the proportion in fecal isolates at 13% (P = 0.02).

### Distribution of VF genes in *E. coli* phylogenetic group B2 isolates from women and children vs. ST131 status

**Fig. 1:**
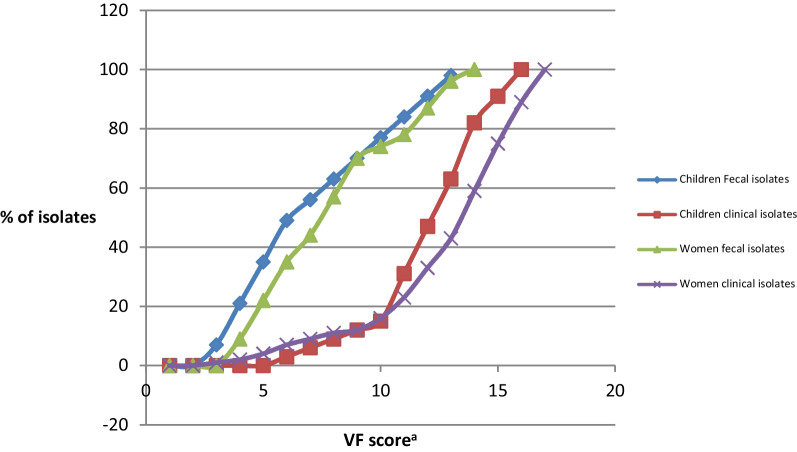
Cumulative frequency of VF scores for *E. coli* isolates from women and children. VF score^a^: The sum of the individual VF genes in an isolate

- Women
  Our second objective was to compare the inferred virulence potential of ST131 vs. non-ST131 isolates within phylogenetic group B2. Thus, non-ST131 B2 fecal and UTI isolates from each host group, collected from the same location and time period, were analyzed in relation to ST131 isolates. However, to reduce duplication of results, data analysis from isolates from men was not included as the results were generally similar to those of women. Overall, based on inferred virulence potential as per Johnson et al. (2006) study [14], within each source group in the women isolates, ST131 isolates had, on average, a higher inferred virulence potential than non-ST131 for most VF genes studied (Table 2). Within sub-group comparisons by ST131 status showed that on average, UTI isolates had higher prevalence for most VF genes, compared to fecal isolates. Specifically, 13 out of 22 VF genes were much more frequent in UTI than fecal isolates. Accordingly, VF gene scores were significantly higher for UTI than fecal isolates in each subgroup (P < 0.001) (Fig. 1). Six VF genes (*fimH*, *focG*, *iutA*, *traT*, *ompT*, *usp*) were detected in all ST131 isolates irrespective of source.
- Children
  The distribution of VF genes in ST131 vs. non-ST131 phylogenetic group B2 isolates from children was similar to that in women and men, with higher prevalence of most VF genes in ST131 UTI isolates (compared to fecal ones) (Table 3). Specifically, among ST131 isolates, 10 out of 22 VF genes were much more frequent in UTI than fecal isolates, whilst in only 2 of 22 VF genes, it was in favor of fecal isolates. Likewise, among non-ST131 isolates, 10 of 22 VF genes were much more abundant in UTI than fecal isolates. Accordingly, in each subgroup, VF gene scores were significantly higher for UTI than fecal isolates (P < 0.001) (Fig. 1).
  Further VF gene score analysis by source and ST131 status revealed that fecal ST131 and fecal non-ST131 B2 isolates were much closer in inferred virulence potential than were the UTI ST131 and non-ST131 isolates. However, fecal isolates from women (ST131 vs. non-ST131 B2) differed much more in inferred virulence potential (as measured by the VF gene scores) to the fecal isolates in children (P = 0.01) (Fig. 1). Likewise, UTI isolates (ST131 vs. non-ST131) from children differed much more than their women counterparts, with isolates from children appearing much closer in virulence potential than those from women. Specifically in UTI isolates from women, the prevalence of most VF genes was almost doubled in ST131 than in non-ST131 isolates.

**Table 2:** Distribution of VF genes in *E. coli* phylogenetic group B2 isolates from women by ST131 status

VF genes^b^	Number (%) of isolates with trait by source and ST131 status				P value by isolate source (vs. ST131)^a^	
	Fecal (n = 101)		Clinical (n = 398)			
	ST131 (n = 23)	Non-ST131 (n = 78)	ST131 (n = 124)	Non-ST131 (n = 274)	Fecal	Clinical^c^
*afa/draBC*	5 (22)	10 (13)	23 (19)	25 (9)	< 0.001	< 0.001
*bmaE*	0 (0)	0 (0)	5 (4)	0 (0)	NS	NS
*sfaS*	7 (30)	10 (13)	26 (21)	36 (13)	< 0.001	0.032
*fimH*	23 (100)	73(94)	124 (100)	266 (97)	NS	NS
*focG*	7 (30)	10 (13)	58 (47)	85 (31)	< 0.001	0.016
*papGI*	0 (0)	0 (0)	5 (4)	0 (0)	NS	0.042
*papGII*	5 (22)	13 (17)	47 (38)	66 (24)	0.041	0.031
*papGIII*	5 (22)	14 (18)	51 (41)	69 (25)	0.045	< 0.001
*papAH*	9 (39)	17 (22)	73 (59)	85 (31)	0.033	< 0.001
*papC*	9 (39)	16 (21)	66 (53)	88 (32)	0.02	< 0.001
*papEF*	10 (43)	12 (15)	62 (50)	80 (29)	< 0.001	< 0.001
*gafD*	0 (0)	2 (3)	10 (8)	3 (1)	NS	0.03
*cnf1*	5 (22)	13 (17)	52 (42)	53 (19)	NS	< 0.001
*hlyA*	5 (22)	16 (21)	47 (38)	58 (21)	NS	< 0.001
*iutA*	23 (100)	41 (53)	124 (100)	178 (65)	< 0.001	< 0.001
*fyuA*	22 (95)	34 (44)	124 (100)	198 (72)	< 0.001	< 0.001
*iroN*	12 (52)	24 (31)	110 (89)	168 (61)	< 0.001	< 0.001
*kpsMTII*	5 (22)	15 (19)	75 (60)	140 (51)	NS	NS
*kpsMTIII*	0 (0)	2 (3)	6 (5)	17 (6)	0.041	NS
*traT*	23 (100)	41 (53)	124 (100)	181 (66)	< 0.001	< 0.001
*ompT*	23 (100)	34 (44)	124 (100)	195 (71)	< 0.001	< 0.001
*usp*	23 (100)	56 (72)	124 (100)	198 (72)	< 0.001	< 0.001

^a^P values are shown where P < 0.05, calculated by Fisher’s exact test. NS, non-significant

^b^The 22 virulence factors analyzed were; *papA*, P fimbriae structural subunit; *papC*, P fimbriae assembly; *papEF*, fimbriae tip pilins; *papG,* P fimbriae adhesin (and alleles I, II and III); *sfaS*, S fimbriae; *focG*, F1C fimbriae; *afa/draBC*, Afimbrial adhesin (Dr-binding adhesin); *fimH*, type 1 fimbriae; *hlyA*, hemolysin; *cnf1,* cytotoxic necrotizing factor type1; *fyuA*, ferric yersiniabactin receptor; *iutA*, aerobactin receptor; *iroN*, catecholate siderophore receptor; *kpsMTII* group 2 capsule (with K1 and K2 variants); *kpsMTIII*, group 3 capsule; *traT*, serum-resistance associated; *ompT*, outer membrane protein T (protease); *bmaE*, M fimbriae; *gafD*, (G) fimbriae

^c^Clinical, cystitis and pyelonephritis isolates

**Table 3:** Distribution of VF genes in *E. coli* phylogenetic group B2 isolates from children by ST131 status

VF genes^b^	Number (%) of isolates with trait by source and ST131 status				P value by isolate source (vs. ST131)^a^	
	Fecal (n = 106)		Clinical (n = 237)			
	ST131 (n = 14)	Non-ST131 (n = 92)	ST131 (n = 32)	Non-ST131 (n = 205)	Fecal	^c^Clinical
*afa/draBC*	2 (13)	3 (3)	8 (25)	27 (13)	0.03	0.002
*bmaE*	0 (0)	0 (0)	1 (3)	0 (0)	NS	0.03
*sfaS*	3 (21)	14 (15)	6 (19)	27 (13)	0.01	0.045
*fimH*	14 (100)	91 (99)	32 (100)	201(98)	NS	NS
*focG*	14 (100)	46 (50)	32 (100)	115 (56)	< 0.001	< 0.001
*papGI*	0 (0)	1 (1)	0 (0)	0 (0)	NS	NS
*papGII*	2 (13)	9 (10)	7 (22)	23 (11)	NS	< 0.001
*papGIII*	3 (21)	14 (15)	13 (41)	29 (14)	0.01	< 0.001
*papAH*	4 (29)	19 (21)	13 (41)	27 (13)	0.043	< 0.001
*papC*	4 (29)	17 (18)	15 (47)	37 (18)	0.003	< 0.001
*papEF*	5 (36)	17 (18)	16 (50)	52 (25)	0.004	< 0.001
*gafD*	0 (0)	0 (0)	1 (3)	0 (0)	NS	NS
*cnf1*	3 (21)	14 (15)	10 (31)	44 (21)	0.01	0.01
*hlyA*	3 (21)	8 (9)	10 (31)	37 (18)	0.009	0.003
*iutA*	14 (100)	63 (68)	32 (100)	156 (76)	< 0.001	< 0.001
*fyuA*	10 (71)	30 (33)	32 (100)	132 (64)	< 0.001	< 0.001
*iroN*	8 (57)	30 (33)	20 (63)	78 (38)	< 0.001	< 0.001
*kpsMTII*	3 (21)	5 (5)	32 (100)	0 (0)	< 0.001	< 0.001
*kpsMTIII*	0 (0)	0 (0)	1 (3)	0 (0)	NS	NS
*traT*	14 (100)	63 (68)	32 (100)	152 (74)	< 0.001	< 0.001
*ompT*	14 (100)	62 (67)	32 (100)	150 (73)	< 0.001	< 0.001
*Usp*	14 (100)	59 (64)	32 (100)	162 (79)	< 0.001	< 0.001

^a^P values are shown where P < 0.05, calculated by Fisher’s exact test. NS, non-significant

^b^The 22 virulence factors analyzed were; *papA*, P fimbriae structural subunit; *papC*, P fimbriae assembly; *papEF*, fimbriae tip pilins; *papG,* P fimbriae adhesin (and alleles I, II and III); *sfaS*, S fimbriae; *focG*, F1C fimbriae; *afa/draBC*, Afimbrial adhesin (Dr-binding adhesin); *fimH*, type 1 fimbriae; *hlyA*, hemolysin; *cnf1,* cytotoxic necrotizing factor type1; *fyuA*, ferric yersiniabactin receptor; *iutA*, aerobactin receptor; *iroN*, catecholate siderophore receptor; *kpsMTII* group 2 capsule (with K1 and K2 variants); *kpsMTIII*, group 3 capsule; *traT*, serum-resistance associated; *ompT*, outer membrane protein T (protease); *bmaE*, M fimbriae; *gafD*, (G) fimbriae

^c^Clinical, cystitis and pyelonephritis isolates

### Distribution of VF genes vs. fluoroquinolone (FQ) resistance status among ST131 isolates in women, men and children

- Women and men
  In order to gain further insights into the relationship between FQ resistance and ST131 status, we examined VF gene prevalence among ST131 isolates by FQ susceptibility phenotype, ESBL production, and O antigen type, in women, men and children isolates (Tables 4, 5, 6). All the ST131 fecal isolates from women and men were of serotype O25b, whilst for the UTI isolates 85% (and 82%) and 94% (and 96%) of FQ-R and FQ-S from women (and men) were O25b, with no significant difference between the two (P > 0.05) (Tables 4 and 5). The O16 serotype was confined to UTI isolates only, with the majority (15% for women and 19% for men) confined to the FQ-R subset, compared to only 5% in women and 4% in men for the FQ-S subset (P = 0.02). ESBL production was highest in FQ-R UTI isolates for both men (67%) and women (77%) isolates, intermediate in FQ-S UTI isolates (45% for men and 44% for women) and FQ-R fecal (about 40%), and lowest in fecal FQ-S (0% and 6%), *albeit* very low numbers of feal isolates in each subgroup (P < 0.001).
  Overall, for both women and men, FQ-R ST131 UTI isolates exhibited a significantly higher prevalence of most VF genes than their FQ-S counterparts. For fecal isolates, FQ-S ST131 isolates appeared modestly more virulent than FQ-R ST131, *albeit* small number of isolates, with VF gene scores modestly different (P = 0.02) (Additional file 1: Table S1). However, for UTI isolates, the difference between FQ-R and FQ-S VF gene scores was significantly higher than in fecal isolates for both cohorts. Specifically, for ST131 UTI isolates from both women and men, at least 10 of 22 VF genes were much more abundant in FQ-R UTI isolates than in FQ-S isolates, *albeit* small number of isolates from men in the 2 subsets. And for fecal isolates, significantly higher prevalence of 4 out of 22 VF genes was observed in FQ-S ST131 than in FQ-R ST131 in women isolates. For fecal isolates from men, the fewer number of ST131 isolates made statistically significant comparison impossible, although the 3 FQ-S isolates carried on average more VF genes. Accordingly, VF gene scores for clinical isolates for both women and men were significantly higher for FQ-R than FQ-S, and lower in FQ-R than FQ-S in fecal isolates (Additional file 1: Table S1). Consequently, within the FQ-R ST131 subset, UTI isolates exhibited a greater abundance of VF genes than fecal isolates. In contrast to the UTI isolates situation, the FQ-S isolates differed minimally between the two source groups, slightly in favor of UTI isolates, with no significant statistical difference. VF gene scores were thus not significantly different between the two (data not shown).
- Children
  The distribution of VF genes by FQ phenotype, O antigen type and ESBL production in children was similar to that in women and men, *albeit* very small sample sizes in each subset (Table 5). Differences with isolates from adults included that one isolate of O16 serotype was from fecal FQ-S ST131 subset, and the higher proportion of FQ-R ST131 isolates which were of O16 serotype (33%) compared to 15% in women (P = 0.01). Furthermore, fecal isolates were much closer in inferred virulence potential when compared to their counterparts in adults. Fecal FQ-R isolates contained on average a lower frequency of VF genes compared to the FQ-S. However, FQ-R UTI isolates were slightly more virulent appearing compared to their FQ-S counterparts, with VF gene scores modestly different (P = 0.01).

**Table 4:** Distribution of VF genes in relation to fluoroquinolone resistance status among *E. coli* ST131 isolates from women

Trait	Number (%) of isolates with trait by source and ST131 status				P value by isolate source (vs. ST131)^a^	
	Fecal (n = 23)		Clinical (n = 131)			
	FQ-R-ST131 (n = 7)	FQ-S-ST131 (n = 16)	FQ-R-ST131 (n = 65)	FQ-S-ST131 (n = 59)	Fecal	Clinical^b^
ESBL	3 (42)	1 (6)	50 (77)	26 (44)	< 0.001	< 0.001
O25b	7 (100)	16 (100)	55 (85)	50 (94)	NS	NS
O16	0 (0)	0 (0)	10 (15)	3 (5)	NS	< 0.001
VF genes^c^						
*afa/draBC*	1 (14)	3 (19)	6 (9)	14 (24)	NS	< 0.001
*bmaE*	0 (0)	0 (0)	1 (2)	0 (0)	NS	NS
*sfaS*	1 (14)	3 (19)	7 (11)	14 (24)	NS	< 0.001
*fimH*	7 (100)	16 (100)	65 (100)	59 (100)	NS	NS
*focG*	2 (29)	7 (44)	44 (68)	17 (29)	0.041	< 0.001
*papGI*	0 (0)	0 (0)	0 (0)	1 (2)	NS	NS
*papGII*	1 (14)	4 (25)	25 (38)	10 (17)	0.031	< 0.001
*papGIII*	1 (14)	3 (19)	20 (31)	7 (12)	0.041	< 0.001
*papAH*	2 (29)	7 (44)	46 (71)	19 (32)	0.032	< 0.001
*papC*	2 (29)	5 (31)	44 (68)	21 (36)	NS	< 0.001
*papEF*	2 (29)	4 (25)	44 (68)	19 (32)	NS	< 0.001
*gafD*	0 (0)	0 (0)	1 (2)	0 (0)	NS	NS
*cnf1*	1 (14)	3 (19)	44 (68)	18 (31)	NS	< 0.001
*hlyA*	1 (14)	3 (19)	47 (72)	187(29)	NS	< 0.001
*iutA*	7 (100)	16 (100)	65 (100)	59 (100)	NS	NS
*fyuA*	5 (71)	11 (69)	65 (100)	47 (80)	NS	0.01
*iroN*	3 (43)	6 (38)	57 (88)	25 (42)	NS	< 0.001
*kpsMTII*	1 (14)	5 (31)	33 (51)	59 (100)	0.028	NS
*KpsMTIII*	0 (0)	0 (0)	0 (0)	0 (0)	NS	NS
*traT*	7 (100)	16 (100)	65 (100)	59 (100)	NS	NS
*ompT*	7 (100)	16 (100)	65 (100)	59 (100)	NS	NS
*usp*	7 (100)	16 (100)	65 (100)	59 (100)	NS	NS

ESBL, extended spectrum β-lactamase; FQ-R, fluoroquinolone resistant; FQ-S, fluoroquinolone susceptible

^a^P values are shown where P < 0.05, calculated by Fisher’s exact test. NS, non-significant

^b^Clinical, cystitis and pyelonephritis isolates

^c^The 22 virulence factors analyzed were; *papA*, P fimbriae structural subunit; *papC*, P fimbriae assembly; *papEF*, fimbriae tip pilins; *papG,* P fimbriae adhesin (and alleles I, II and III); *sfaS*, S fimbriae; *focG*, F1C fimbriae; *afa/draBC*, Afimbrial adhesin (Dr-binding adhesin); *fimH*, type 1 fimbriae; *hlyA*, hemolysin; *cnf1,* cytotoxic necrotizing factor type1; *fyuA*, ferric yersiniabactin receptor; *iutA*, aerobactin receptor; *iroN*, catecholate siderophore receptor; *kpsMTII* group 2 capsule (with K1 and K2 variants); *kpsMTIII*, group 3 capsule; *traT*, serum-resistance associated; *ompT*, outer membrane protein T (protease); *bmaE*, M fimbriae; *gafD*, (G) fimbriae

**Table 5:** Distribution of VF genes in relation to fluoroquinolone (FQ) resistance status among *E. coli* ST131 isolates from men

Trait	Number (%) of isolates with trait by source and ST131 status				P value by isolate source (vs. ST131)^a^	
	Fecal (n = 8)		Clinical (n = 49)			
	FQ-R-ST131 (n = 5)	FQ-S-ST131 (n = 3)	FQ-R-ST131 (n = 27)	FQ-S-ST131 (n = 22)	Fecal	Clinical^b^
ESBL	2 (40)	0 (0)	18 (67)	10 (45)	< 0.001	< 0.001
O25b	5 (100)	3 (100)	22 (82)	21 (96)	NS	NS
O16	0 (0)	0 (0)	5 (19)	1 (4)	NS	< 0.001
VF genes^c^						
*afa/draBC*	1 (20)	0 (0)	1 (4)	4 (18)	NS	< 0.001
*bmaE*	0 (0)	0 (0)	1 (4)	0 (0)	NS	NS
*sfaS*	1 (20)	0 (0)	2 (7)	5 (23)	NS	< 0.001
*fimH*	5 (100)	3 (100)	27 (100)	22 (100)	NS	NS
*focG*	1 (20)	1 (33)	18 (67)	6 (27)	0.031	< 0.001
*papGI*	0 (0)	0 (0)	0 (0)	0 (0)	NS	NS
*papGII*	1 (20)	1 (33)	8 (30)	3 (14)	0.031	< 0.001
*papGIII*	1 (20)	1 (33)	8 (30)	4 (18)	0.039	< 0.001
*papAH*	1 (20)	1 (33)	16 (59)	6 (27)	0.030	< 0.001
*papC*	1 (20)	1 (33)	15 (56)	7 (32)	NS	< 0.001
*papEF*	1 (20)	1 (33)	16 (59)	7 (32)	NS	< 0.001
*gafD*	0 (0)	0 (0)	1 (4)	0 (0)	NS	NS
*cnf1*	1 (20)	1 (33)	17 (63)	8 (36)	NS	< 0.001
*hlyA*	1 (20)	1 (33)	18 (67)	6 (27)	NS	< 0.001
*iutA*	5 (100)	3 (100)	27 (100)	22 (100)	NS	NS
*fyuA*	3 (60)	2 (67)	27 (100)	16 (73)	NS	0.01
*iroN*	2 (40)	1 (33)	22 (81)	9 (41)	NS	< 0.001
*kpsMTII*	1 (20)	1 (33)	13 (48)	22 (100)	0.027	NS
*KpsMTIII*	0 (0)	0 (0)	0 (0)	0 (0)	NS	NS
*traT*	5 (100)	3 (100)	27 (100)	22 (100)	NS	NS
*ompT*	5 (100)	3 (100)	27 (100)	22 (100)	NS	NS
*usp*	5 (100)	3 (100)	27 (100)	22 (100)	NS	NS

ESBL, extended spectrum β-lactamase; FQ-R, fluoroquinolone resistant; FQ-S, fluoroquinolone susceptible

^a^P values are shown where P < 0.05, calculated by Fisher’s exact test. NS, non-significant

^b^Clinical, cystitis and pyelonephritis isolates

^c^The 22 virulence factors analyzed were; *papA*, P fimbriae structural subunit; *papC*, P fimbriae assembly; *papEF*, fimbriae tip pilins; *papG,* P fimbriae adhesin (and alleles I, II and III); *sfaS*, S fimbriae; *focG*, F1C fimbriae; *afa/draBC*, Afimbrial adhesin (Dr-binding adhesin); *fimH*, type 1 fimbriae; *hlyA*, hemolysin; *cnf1,* cytotoxic necrotizing factor type1; *fyuA*, ferric yersiniabactin receptor; *iutA*, aerobactin receptor; *iroN*, catecholate siderophore receptor; *kpsMTII* group 2 capsule (with K1 and K2 variants); *kpsMTIII*, group 3 capsule; *traT*, serum-resistance associated; *ompT*, outer membrane protein T (protease); *bmaE*, M fimbriae; *gafD*, (G) fimbriae

**Table 6:** Distribution of VF genes in relation to fluoroquinolone resistance status among *E. coli* ST131 isolates from children

Trait	Number (%) of isolates with trait by source and ST131 status				P value by isolate source (FQ-R vs. FQ-S)^a^	
	Fecal (n = 14)		Clinical (n = 32)			
	FQ-R-ST131 (n = 5)	FQ-S-ST131 (n = 9)	FQ-R-ST131 (n = 15)	FQ-S-ST131 (n = 17)	Fecal	Clinical^b^
ESBL	2 (40)	1 (11)	8 (53)	5 (29)	0.032	< 0.001
O25b	5 (100)	8 (89)	10 (67)	16 (94)	NS	< 0.001
O16	0 (0)	1 (11)	5 (33)	1 (6)	0.04	0.01
VF genes^c^						
*afa/draBC*	1 (20)	1 (11)	1(7)	3 (18)	NS	0.02
*bmaE*	0 (0)	0 (0)	0 (0)	1 (6)	NS	NS
*sfaS*	1 (20)	3 (33)	2 (7)	3 (18)	0.04	0.023
*fimH*	4 (80)	9 (100)	15 (100)	17 (100)	0.04	NS
*focG*	2 (40)	3 (33)	9 (60)	8 (47)	NS	0.03
*papGI*	0 (0)	0 (0)	0 (0)	1 (6)	NS	NS
*papGII*	1 (20)	4 (44)	4 (27)	4 (24)	0.036	NS
*papGIII*	1 (20)	4 (44)	5 (33)	4 (24)	0.036	0.041
*papAH*	2 (40)	4 (44)	9 (60)	5 (29)	NS	0.01
*papC*	2 (40)	4 (44)	8 (53)	3 (18)	NS	< 0.001
*papEF*	2 (40)	4 (44)	9 (60)	4 (24)	NS	0.01
*gafD*	0 (0)	0 (0)	0 (0)	1 (6)	NS	0.041
*cnf1*	2 (40)	6 (67)	9 (60)	6 (35)	0.035	0.021
*hlyA*	2 (40)	6 (67)	9 (60)	6 (35)	0.035	0.021
*iutA*	4 (80)	9 (100)	15 (100)	17 (100)	0.036	NS
*fyuA*	2 (40)	9 (100)	15 (100)	9 (53)	0.035	0.031
*iroN*	2 (40)	6 (67)	11 (73)	8 (47)	0.033	0.01
*kpsMTII*	1 (20)	6 (67)	8 (47)	10 (58)	0.023	0.043
*kpsMTIII*	0 (0)	0 (0)	1 (7)	0 (0)	NS	0.037
*traT*	5 (100)	9 (100)	15 (100)	17 (100)	NS	NS
*ompT*	5 (100)	9 (100)	15 (100)	17 (100)	NS	NS
*usp*	5 (100)	9 (100)	15 (100)	17 (100)	NS	NS

ESBL, extended spectrum β-lactamase; FQ-R, fluoroquinolone resistant; FQ-S, fluoroquinolone susceptible

^a^P values are shown where P < 0.05, calculated by Fisher’s exact test. NS, non-significant

^b^Clinical, cystitis and pyelonephritis isolates

^c^The 22 virulence factors analyzed were; *papA*, P fimbriae structural subunit; *papC*, P fimbriae assembly; *papEF*, fimbriae tip pilins; *papG,* P fimbriae adhesin (and alleles I, II and III); *sfaS*, S fimbriae; *focG*, F1C fimbriae; *afa/draBC*, Afimbrial adhesin (Dr-binding adhesin); *fimH*, type 1 fimbriae; *hlyA*, hemolysin; *cnf1,* cytotoxic necrotizing factor type1; *fyuA*, ferric yersiniabactin receptor; *iutA*, aerobactin receptor; *iroN*, catecholate siderophore receptor; *kpsMTII* group 2 capsule (with K1 and K2 variants); *kpsMTIII*, group 3 capsule; *traT*, serum-resistance associated; *ompT*, outer membrane protein T (protease); *bmaE*, M fimbriae; *gafD*, (G) fimbriae

### Distribution of VF genes by fluoroquinolone (FQ) susceptibility pattern and ESBL status among *E. coli* ST131 isolates from women, men and children

To identify possible reasons for ST131’s rapid spread, ESBL and VF gene profiles were examined in relation to FQ susceptibility phenotype (Tables 6 and 7). Due to small sample sizes in each subgroup, we combined all the isolates from women, men and children, in order to have reasonable sample sizes for statistical analysis, and also only analyzed UTI isolates. Overall, FQ-R ESBL isolates harboured a greater proportion of VF genes compared to the other 3 subsets, with 10/22 VF genes having at least double frequencies in this subset compared to others (Table 7). Consequently, VF gene scores were highest in this subset. Within the FQ-R subset, ESBL positive isolates showed significantly higher VF gene scores compared to non-ESBL (P < 0.001) isolates (Additional file 1: Table S2). However, for the FQ-S subset, the results were mixed between ESBL and non-ESBL, with some VF genes more prevalent in the ESBL subset and others in the non-ESBL subset. Accordingly, VF gene scores were generally similar between the ESBL and non-ESBL FQ-S isolates (P > 0.05). All the rare VF genes (*bmaE*, *gafD*, *papGI*) were confined to the FQ-R ESBL subset.

**Table 7:** Distribution of VF genes in relation to fluoroquinolone resistance status and ESBL among *E. coli* ST131 clinical isolates from women, men and children

VF genes^b^	Number (%) of isolates with trait by source and ST131 status				P value by isolate source (ESBL vs. non-ESBL)^a^	
	FQ-R (n = 142)		FQ-S (n = 131			
	ESBL (n = 105)	Non-ESBL (n = 37)	ESBL (n = 53)	Non-ESBL (n = 78)	FQ-R	FQ-S
*afa/draBC*	13 (12)	14 (38)	10 (19)	31 (40)	< 0.001	< 0.001
*bmaE*	2 (2)	0 (0)	0 (0)	0 (0)	NS	ns
*sfaS*	17 (16)	13 (36)	10 (19)	31 (40)	< 0.001	0.004
*fimH*	105 (100)	37 (100)	53 (100)	78 (100)	NS	NS
*focG*	85 (81)	22 (59)	31 (58)	33 (42)	0.003	0.023
*papGI*	2 (2)	0 (0)	0 (0)	0 (0)	NS	NS
*papGII*	70 (67)	22 (59)	15 (29)	13 (16)	0.032	< 0.001
*papGIII*	60 (57)	15 (41)	9 (17)	16 (20)	0.04	NS
*papAH*	76 (72)	12 (32)	17 (32)	26 (33)	< 0.001	NS
*papC*	72 (69)	19 (50)	19 (35)	30 (38)	0.003	NS
*papEF*	82 (78)	22 (59)	15 (29)	31 (40)	< 0.001	< 0.001
*gafD*	2 (2)	0 (0)	0 (0)	0 (0)	NS	NS
*cnf1*	70 (67)	20 (55)	28 (52)	30 (38)	0.002	< 0.001
*hlyA*	76 (72)	15 (41)	22 (42)	23 (29)	< 0.001	< 0.001
*iutA*	105 (100)	37 (100)	53 (100)	78 (100)	NS	NS
*fyuA*	105 (100)	37 (100)	53 (100)	78 (100)	NS	NS
*iroN*	18 (17)	7 (18)	7 (13)	3 (4)	NS	0.031
*kpsMTII*	105 (100)	37 (100)	53 (100)	78 (100)	NS	NS
*kpsMTIII*	2 (2)	0 (0)	0 (0)	0 (0)	NS	NS
*traT*	105 (100)	22 (100)	53 (100)	78 (100)	NS	NS
*ompT*	105 (100)	22 (100)	53 (100)	78 (100)	NS	NS
*usp*	105 (100)	22 (100)	53 (100)	78 (100)	NS	NS

ESBL, extended spectrum β-lactamase; FQ-R, fluoroquinolone resistant; FQ-S, fluoroquinolone susceptible

^a^P values are shown where P < 0.05, calculated by Fisher’s exact test. NS, non-significant

^b^The 22 virulence factors analyzed were; *papA*, P fimbriae structural subunit; *papC*, P fimbriae assembly; *papEF*, fimbriae tip pilins; *papG,* P fimbriae adhesin (and alleles I, II and III); *sfaS*, S fimbriae; *focG*, F1C fimbriae; *afa/draBC*, Afimbrial adhesin (Dr-binding adhesin); *fimH*, type 1 fimbriae; *hlyA*, hemolysin; *cnf1,* cytotoxic necrotizing factor type1; *fyuA*, ferric yersiniabactin receptor; *iutA*, aerobactin receptor; *iroN*, catecholate siderophore receptor; *kpsMTII* group 2 capsule (with K1 and K2 variants); *kpsMTIII*, group 3 capsule; *traT*, serum-resistance associated; *ompT*, outer membrane protein T (protease); *bmaE*, M fimbriae; *gafD*, (G) fimbriae

## Discussion

Despite an increase in the number of studies on *E. coli* ST131, a lot still remains unknown about this organism, especially characteristics that promote its rapid worldwide spread. We studied the distribution of ST131 by source (fecal vs. UTI), host group (women, men and children), fluoroquinolone (FQ) resistance phenotype, and B2 phylogenetic group status, among *E. coli* isolates from the Central West region of NSW, Australia. We found out that there was a step-up in ST131 prevalence (and possibly inferred virulence potential) from fecal to UTI isolates in general, and specifically, among ciprofloxacin resistant isolates in the 3 host groups, with the trend more marked in isolates from women.

These findings suggest that there is diversity within the ST131 group, with isolates from feces appearing less virulent than UTI isolates. Assuming that the fecal flora is the major reservoir of ST131 and other UPEC strains, these findings further suggest that acquisition of other genetic elements linked to pathogenesis may be important in ST131 transmission [12]. For UTI pathogenesis, these findings support the special pathogenicity theory, which posits that UTI occurs when *E. coli* strains with special factors move from fecal flora to the urinary tract [15].

The second part of our study was to gain further insights into the inferred virulence potential (based on VF gene scores) of *E. coli* ST131 compared to non-ST131 isolates within phylogenetic group B2. Previous studies have compared ST131 with other non-ST131 strains without taking into consideration the possible confounding effect of phylogenetic groups on VF genes, especially for fecal isolates in which the majority of isolates are likely to be of other phylogenetic groups other than B2 [16–18]. Even in clinical isolates, about 34% of non-ST131 isolates are of other phylogenetic groups other than B2 [15].

Our results revealed that ST131 isolates from both adults and children had higher inferred virulence potential than their non-ST131 counterparts within phylogenetic group B2, which is known to harbor more VF genes than other phylogenetic groups [19]. This finding suggests possible presence of B2 subgroups with various levels of inferred virulence, with ST131 being highly virulent when compared to others. Thus, the high prevalence of ST131 among our UTI isolates may be explained by the possible high virulence potential of such strains compared to others. These results confirm our previous findings indicating that there are subgroups with differing levels of virulence potential within group B2 [20]. Indeed, Le Gall et al. [21] identified nine subgroups within group B2, and Clermont et al. (2009) placed *E. coli* ST131 in subgroup I, which was suggested to be the basal subgroup of B2 strains [22]. In addition, in the present study, isolates from women and men (ST131 vs. non-ST131) differed significantly for VF gene scores within each subset to those in children, which appeared much closer in inferred virulence potential than in isolates from adults. This finding also suggests presence of ST131 pathotypes adapted to specific hosts depending on several host factors. It is possible that the low immunity levels in children expose them to infection with less virulent ST131 strains compared to adults.

Apparently, the present findings reflecting higher VF gene carriages amongst ST131 strains contradict other studies where the ST131 strains exhibited lower VF gene scores compared to other non-ST131 strains [23, 24], but also confirm findings from others [25, 26]. Possible explanations for these differences include the confounding effect of other phylogenetic groups other than B2, VF gene distribution differences due to geographical location, and the number of isolates tested. Thus, it is important that comparisons between studies take into consideration the source and host group of the isolates in pathogenesis studies, including the in vivo animal studies.

Several animal studies have been carried out to understand the pathogenesis of ST131, with contradicting outcomes especially in relation to inferred virulence potential as measured by VF gene scores. Animal studies by Lavigne et al. (2012), assessing the virulence potential of 3 ST131 and 5 non-ST131 phylogenetic B2 strains in two animal models, showed that ST131 strains were less virulent than non-ST131 in the nematode model [27]. Such findings must be interpreted with caution because of the smaller number of isolates tested, and the physiological differences between nematodes and humans. Furthermore, mouse model studies have also yielded conflicting results, resulting in some researchers, including Johnson and colleagues, to conclude that ST131 strains have broad virulence diversity in the mouse sepsis model [28, 29]. The present results seem to support this conclusion.

Despite most of ST131’s enhanced virulence potential being likely driven by defined virulence traits, it is plausible that other unknown B2-associated and non-B2 characteristics, are involved. Indeed, some researchers have demonstrated that other yet to be defined group B2 associated factors may provide a fitness advantage to ST131 [30], however other studies did not find any difference in the metabolic activity of ST131 vs. no-ST131 strains [31]. It would be interesting to determine the metabolic activity of ST131 vs. non-ST131 B2 from the same locale and time period, to confirm this theory.

The present study revealed the high virulence potential of ST131 O16 isolates which were overwhelmingly confined to FQ-R UTI isolates in the 3 host groups at 15% for women, 19% for man and 33% for children, with only one fecal isolate confined to FQ-S in children. The small sample size of O16 isolates (n = 26), limited statistical power for further assessments. However, VF gene scores for each of the O16 isolates was at least 15 (data not shown), suggesting heightened virulence potential for such isolates. However, Mora et al. (2014) reported low VF gene scores for O16:H5 isolates but with similar mouse killing capacity to that of highly virulent 025:H4 strains [28]. Further studies with large sample sizes are needed to confirm these findings.

The strong association between ST131 prevalence and FQ resistance amongst the studied isolates suggests that FQ use is related to ST131 emergence and spread. Specifically, 78% of the FQ-R UTI isolates from women, 77% in men, and 47% from children, were ESBL producers. Moreover, FQ-R ST131 clinical ESBL producing isolates harbored on average more VF genes than all other isolates. Thus, the present study demonstrates that FQ-resistance and a plurality of VF genes can exist together, something that has traditionally been regarded as inversely related [32]. These findings confirm, from a different population and geographical location, previous findings [26, 27] and provide possible explanation for the remarkable success of ST131 as a pathogen, including group B2 phylogenetic background, plurality of VF genes and antimicrobial resistance [2].

The present findings on the distribution of VF genes by FQ phenotype and ESBL production also suggest the presence of subgroups within ST131 with differing levels of virulence. The combined characteristics of FQ-R, ESBL production and high VF scores in ST131 isolates may give such isolates a fitness advantage of other strains, and promote their rapid and world-wide spread. These findings establish ST131 as an important emerging public health threat in Australia requiring urgent surveillance so that appropriate control measures can be devised.

Previous studies have revealed that FQ resistance is associated with the entire *H*30 subclone, whilst ESBL production and CTX-M-15 are associated specifically with the *H*30-Rx subset [33–36]. We did not characterize our isolates for the H30-R and H30-Rx sublineages, but speculate that the majority of the FQ-R ESBL might belong to the H30-R sublineage, which in a study in Canada accounted for 71% of the FQ-R and 62% of the ESBL-producing isolates. Interestingly, a study by Banerjee et al. in the Chicago region of USA demonstrated that H30-R and H30-Rx were more antimicrobial resistant, and the H30-Rx sublineage had the highest virulence gene scores, implying greater virulence potential and possibly explaining its high prevalence [33].

Our study was aimed at comparing VF genes between ST131 and non-ST131 within phylogenetic group B2. Thus, we did not include detailed antimicrobial resistance data. However, overall, the ST131 isolates had higher prevalence rates of antimicrobial resistance amongst the antibiotics tested, than their non-ST131 B2 counterparts (data not shown), a finding consistent with previous findings [36]. Thus, ST131 appears to combine both antimicrobial resistance and virulence, which in *E. coli* traditionally have been somewhat mutually exclusive [37–39].

The strengths of this study include the large sample size of well characterized isolates from women, men and children, collected consecutively over 3 years and from the same geographical location. Weaknesses of the study include the small sample sizes of some sub-groups, which limited statistical power. We also arbitrarily chose 10 colonies for each rectal swab culture, which would have diminished the chances of picking up ST131 as a dominant clone. The use of multiple comparisons increased the likelihood of finding differences by chance alone (type 2 error), and the use of a 10 µg ciprofloxacin disc may have compromised picking all the resistant isolates. In addition, we did not characterize further the ST131 isolates into distinct subclones which would probably have yielded further information. And finally, the reference to virulence potential in this study is inferred from VF gene scores, and may thus not be reflective of the actual virulence level of the organism in vivo. However, VF gene scores have been shown to be predictive of virulence based on previous in vivo studies in animal models [14, 28], *albeit* mouse models that are more suited to studying sepsis rather than UTI.

## Conclusions

Our study findings suggest a possible step up in inferred virulence potential in *E. coli* strains from fecal isolates to those derived from UTI cases, and this trend was specifically demonstrated among ciprofloxacin resistant isolates, suggesting that the success of *E. coli* ST131 and its worldwide spread may be due to antibiotic resistance and carriage of a wide range of VF genes. However, these findings are considered preliminary and await confirmation in future studies.

## Supplementary Information

**Additional file 1: Table 1.** Distribution ofvirulence scores by source and fluoroquinolone (FQ) resistance phenotypeamongst* Escherichia coli* ST131isolates from children and women.

## Data Availability

The datasets and range of bacterial isolates analysed are available from the corresponding author upon reasonable request.

## References

[1] Medina M, Castillo-Pino E (2019). An introduction to the epidemiology and burden of urinary tract infections. Ther Adv Urol.

[2] Hojabri Z, Darabi N, Arab M, Saffari F, Pajand O. Clonal diversity, virulence genes content and subclone status of *Escherichia coli* sequence type 131: comparative analysis of *E. coli* ST131 and non-ST131 isolates from Iran. BMC Microbiol. 2019; 117. 10.1186/s12866-019-1493-8 PMC6543562 31146674

[3] Biggs M, Moons P, Nguyen MN, Goossens H, Van Puyvelde S (2022). Convergence of virulence and antimicrobial resistance in increasingly prevalent *Escherichia coli* ST131 *papGII*+ sublineages. Commun Biol.

[4] Ben Zakour NL, Alsheikh-Hussain AS, Ashcroft MM, Khanh Nhu NT, Roberts LW, Stanton-Cook M, Schembri MA, Beatson SA. Sequential acquisition of virulence and fluoroquinolone resistance has shaped the evolution of *Escherichia coli* ST131**.** mBio. 2021; 12:e0145121. 10.1128/mBio.00347-16 PMC4850260 27118589

[5] Lidin-Janson G, Kaijser B, Lincoln K, Olling S, Wedel H (1978). The homogeneity of the fecal coliform flora of normal school-girls, characterized by serological and biochemical properties. Med Microbiol Immunol.

[6] Clinical and Laboratory Standards Institute (CLSI) (2012). Performance Standards for Antimicrobial Susceptibility Testing, 17th informational supplement, M100-s17:1–177. Wayne, PA: Clinical and Laboratory Standards Institute.

[7] Sageerabanoo S, Malini A, Mangaiyarkarasi T, Hemalatha G (2015). Phenotypic detection of extended spectrum β-lactamase and Amp-C β-lactamase producing clinical isolates in a tertiary care hospital: a preliminary study. J Nat Sci Biol Med.

[8] Clermont O, Christenson JK, Denamur E, Gordon DM (2013). The Clermont *Escherichia coli* phylo-typing method revisited: improvement of specificity and detection of new phylo-groups. Environ Microbiol Rep.

[9] Clermont O, Johnson JR, Menard M, Denamur E (2007). Determination of *Escherichia coli* O types by allele-specific polymerase chain reaction: application to the O types involved in human septicemia. Diagn Microbiol Infect Dis.

[10] Platell JL, Cobbold RN, Johnson JR, Heisig A, Heisig P, Clabots C, Kuskowski MA, Trott DJ (2011). Commonality among fluoroquinolone resistant sequence type ST131 extraintestinal *Escherichia coli* isolates from humans and companion animals in Australia. Antimicrob Agents Chemother.

[11] Clermont O, Dhanji H, Upton M (2009). Rapid detection of the O25b-ST131 clone of *Escherichia coli* encompassing the CTX-M-15-producing strains. J Antimicrob Chemother.

[12] Johnson JR, Menard M, Johnston B, Kuskowski MA, Nichol K, Zhanel GG (2009). Epidemic clonal groups of *Escherichia coli* as a cause of antimicrobial-resistant urinary tract infections in Canada, 2002 to 2004. Antimicrob Agents Chemother.

[13] Kudinha T, Kong F, Johnson JR, Andrew SD, Anderson P, Gilbert GL (2012). Multiplex PCR-based reverse line blot assay for simultaneous detection of 22 virulence genes in uropathogenic *Escherichia coli*. Appl Environ Microbiol.

[14] Johnson JR, Clermont O, Menard M, Kuskowski MA (2006). Experimental mouse lethality of *Escherichia coli* isolates, in relation to accessory traits, phylogenetic groups and ecological source. J Infect Dis.

[15] Gonzales Decano A, Downing T (2019). An *Escherichia coli* ST131 pangenome atlas reveals population structure and evolution across 4071 isolates. Sci Rep.

[16] Mann R, Mediat DG, Duggin IG, Harry EJ, Bottomley AL (2017). Metabolic adaptations of Uropathogenic *E. coli* in the urinary tract. Front Cell Infect Microbiol.

[17] Halaji M, Fayyazi A, Rajabnia M, Zare D, Pournajaf A, Ranjbar R (2022). Phylogenetic group distribution of uropathogenic *Escherichia coli* and related antimicrobial resistance pattern: a meta-analysis and systematic review. Front Cell Infect Microbiol.

[18] Dadi BR, Abebe T, Zhang L, Mihret A, Abebe W, Amogne W (2020). Distribution of virulence genes and phylogenetics of uropathogenic *Escherichia coli* among urinary tract infection patients in Addis Ababa, Ethiopia. BMC Infect Dis.

[19] Mamani R, Flament-Simon SC, Garcia V (2019). Sequence types, clonotypes, serotypes, and virotypes of extended-spectrum β-lactamase-producing *Escherichia coli* causing bacteraemia in a Spanish hospital over a 12-year period (2000 to 2011). Front Microbiol.

[20] Kudinha T, Johnson JR, Andrew SD, Kong F, Anderson P, Gilbert GL (2013). Distribution of phylogenetic groups, sequence type ST131, and virulence- associated traits among *Escherichia coli* isolates from men with pyelonephritis or cystitis and healthy controls. Clin Microbiol Infect J.

[21] Le Gall T, Clermont O, Gouriou S (2007). Extraintestinal virulence is a coincidental by-product of commensalism in B2 phylogenetic group *Escherichia coli* strains. Mol Biol Evol.

[22] Cummins EA, Snaith AE, McNally A, Hall RJ. The role of potentiating mutations in the evolution of pandemic *Escherichia coli* clones. Europ J Clin Microbiol Infect Dis. 2021; 10.1007/s10096-021-04359-3 34787747

[23] Kondratyeva K, Salmon-Divon M, Navon-Venezia S (2020). Meta-analysis of pandemic *Escherichia coli* ST131 plasmidome proves restricted plasmid-clade associations. Sci Rep.

[24] Hojabri Z, Darabi N, Arab M, Saffari F, Pajand O (2019). Clonal diversity, virulence genes content and subclone status of *Escherichia coli* sequence type 131: comparative analysis of *E. coli* ST131 and non-ST131 isolates from Iran. BMC Microbiol.

[25] Kim B, Kim JH, Lee Y (2022). Virulence factors associated with *Escherichia coli* bacteremia and urinary tract infection. Ann Lab Med.

[26] Ali I, Rafaque Z, Ahmd I, Tariq F, Graham SE, Salzman E, Foxman B, Dasti JI (2019). Phylogeny, sequence-typing and virulence profile of uropathogenic *Escherichia coli* (UPEC) strains from Pakistan. BMC Infect Dis.

[27] Lavigne JP, Vergurot AC, Goret L (2012). Virulence potential and genomic mapping of the worldwide clone *Escherichia coli* ST131. PLoS ONE.

[28] Mora A, Dahbi G, Lopez C, Mamani R (2014). Virulence patterns in a murine sepsis model of ST131 *Escherichia coli* clinical isolates belonging to serotypes O25b:H4 and O16:H5 are associated to specific virotypes. PLoS ONE.

[29] Merion I, Porter SB, Johnston B, Clabots C, Thuras P (2020). Molecularly defined extraintestinal pathogenic *Escherichia coli* status predicts virulence in a murine sepsis model better than does virotype, individual virulence genes, or clonal subset among *E. coli* ST131 isolates. Virulence..

[30] Wu J, Lan F, Lu Y, He Q, Li B (2017). Molecular characteristics of ST1193 clone among phylogenetic group B2 non-ST131 fluoroquinolone-resistant *Escherichia coli*. Front Microbiol.

[31] Alqasim A, Jaffal AA, Almutairi N, Alyousef AA (2021). Comparative phenotypic characterization identifies few differences in the metabolic capacity between *Escherichia coli* ST131 subclones. Saudi J Biol Sci.

[32] Rozwadowski M, Gawel D (2022). Molecular factors and mechanisms driving multidrug resistance in uropathogenic *Escherichia coli*-An update. Genes.

[33] Banerjee R, Strahilevitz J, Johnson JR (2013). Predictors and molecular epidemiology of community-onset extended-spectrum beta-lactamase-producing *Escherichia coli* infection in a Midwestern community. Infect Control Hosp Epidemiol.

[34] Alqasim A, Jaffal AA, Alyousef AA (2020). Prevalence and molecular characteristics of sequence type 131 clone among clinical uropathogenic *Escherichia coli* isolates in Riyadh, Saudi Arabia. Saudi J Biol Sci.

[35] Johnson JR, Porter S, Thuras P, Castanheira M. The Pandemic H30 subclone of sequence type 131 (ST131) as the leading cause of multidrug-resistant *Escherichia coli* infections in the United States (2011–2012). Open Forum Infect Dis 2017; 4:10.1093/ofid/ofx089 PMC5473367 28638846

[36] Johnson JR, Johnston B, Kuskowski MA, Sokurenko EV, Tchesnokova V (2015). Intensity and mechanisms of fluoroquinolone resistance within the H30 and H30Rx subclones of *Escherichia coli* Sequence Type 131 compared with other fluoroquinolone-resistant *E. coli*. Antimicrob Agents Chemother.

[37] Braz VS, Melchior K, Moreira CG (2020). *Escherichia coli* as a multifaceted pathogenic and versatile bacterium. Front Cell Infect Microbiol.

[38] Hastak P, Fourment M, Darling AR, Gottlieb T (2020). *Escherichia coli* ST8196 is a novel, locally evolved, and extensively drug resistant pathogenic lineage within the ST131 clonal complex. Emerg Microbes Infect.

[39] Pitout JDD, DeVinney R (2017). *Escherichia coli* ST131: a multidrug-resistant clone primed for global domination. F1000Research..

