# Supplementary material for: Possible step-up in prevalence for Escherichia coli ST131 from fecal to clinical isolates: inferred virulence potential comparative studies within phylogenetic group B2
Source: J Biomed Sci. 2022 Oct 7;29:78. doi: 10.1186/s12929-022-00862-7 (PMC9547475; doi:10.1186/s12929-022-00862-7)
Supplement: Supplementary file 1 — Additional file 1: Table 1. Distribution ofvirulence scores by source and fluoroquinolone (FQ) resistance phenotypeamongst Escherichia coli ST131isolates from children and women. [file 12929_2022_862_MOESM1_ESM.docx]

**Supplementary Table 1. Distribution of virulence scores by source and fluoroquinolone (FQ) resistance phenotype amongst *Escherichia coli* ST131 isolates from children and women**

| Subset | Children  Median score (range) | Women  Median score (range) |
| --- | --- | --- |
| Fecal FQ-R | 5 (3-8) | 8 (4-12) |
| Fecal FQ-S | 6 (2-9) | 6 (3-8) |
| Clinical FQ-R | 10 (8-15) | 12 (9-17) |
| Clinical FQ-S | 8 (9-14) | 9 (8-15) |

**Note.** FQ-R, fluoroquinolone resistant; FQ-S, fluoroquinolone susceptible.

P values by Mann-Whitney U test where P < 0.05.

NS, not significant (P ≥ 0.05).

**Supplementary Table S2. Distribution of Virulence Scores of *Escherichia coli* ST131 by fluoroquinolone resistance and ESBL production among clinical isolates from children and women.**

| Subset | Number of isolates | Virulence score, median (range) |
| --- | --- | --- |
| FQ-R ESBL | **58** | **12 (10-17)** |
| FQ-R non-ESBL | **22** | **9 (9-16)** |
| FQ-S ESBL | **31** | **7 (6-14)** |
| FQ-S non-ESBL | **45** | **7 (3-12)** |

**Note. ESBL, extended spectrum β-lactamase; FQ-R, fluoroquinolone resistant; FQ-S, fluoroquinolone susceptible.**
